# Three-Dimensional Laparoscopic Varicocelectomy as the New Microsurgical Technique: Safety, Efficiency, and Outcomes

**DOI:** 10.7759/cureus.100148

**Published:** 2025-12-26

**Authors:** Filippos Nikitakis, Nikolaos Grivas, Christos Zabaftis, Maria Chalkidou, Athanasios Bouchalakis, Smaragda Tsela, Markos Karavitakis

**Affiliations:** 1 2nd Department of Urology, Lefkos Stavros - The Athens Clinic, Athens, GRC

**Keywords:** laparoscopic varicocelectomy, male infertility, minimally invasive surgery, three-dimensional laparoscopy, varicocele treatment

## Abstract

Background

Varicocele is a prevalent and potentially correctable cause of male infertility. Surgical intervention is recommended in men with varicocele and abnormal semen parameters. Although three-dimensional (3D) laparoscopy has been widely adopted in urologic surgery, its role in varicocelectomy remains insufficiently investigated.

Objectives

The aim of this study was to evaluate the safety, efficiency, and outcomes of 3D laparoscopic varicocelectomy in a cohort of patients with ultrasound-confirmed varicocele, and to explore whether this approach can serve as a valid alternative to microsurgical varicocelectomy.

Materials and methods

We retrospectively analyzed 34 consecutive patients who underwent 3D laparoscopic varicocelectomy using a standardized approach emphasizing precise anatomical dissection. No adjunctive Doppler or vital dyes were used. Key outcomes included operative time, intraoperative and postoperative complications, recurrence rate, pain scores, patient satisfaction, and semen parameters at six months.

Results

The mean operative time was 22.3 minutes, significantly shorter than reported averages in conventional laparoscopic or microsurgical series. No intraoperative or postoperative complications were recorded, including hydrocele (0%) or testicular atrophy (0%). There was one (1.85%) case of recurrence of varicocele. Postoperative pain was minimal, with a mean visual analog scale (VAS) score of 1.2/10 at 24 hours, and no patients required opioids beyond the first postoperative day. Overall, patient satisfaction was high: 94.1% rated their experience as “very good” or “excellent” at the six-week follow-up. Semen parameters improved significantly: mean sperm concentration increased from 15.1 ×10⁶/mL to 34.3 ×10⁶/mL, and progressive motility from 28.4% to 44.9% (p < 0.01).

Conclusion

Three-dimensional laparoscopic varicocelectomy demonstrates excellent safety, minimal discomfort, high patient satisfaction, and strong functional outcomes. With the advantages of shorter operative time and a low complication rate, it may represent a viable modern alternative to microsurgical varicocelectomy in expert centres.

## Introduction

Varicocele is a common and potentially correctable cause of male infertility, with a prevalence of approximately 15% in the general male population and up to 40% in men with primary infertility [1,2]. It is defined as the abnormal dilatation of the pampiniform venous plexus due to incompetent or absent venous valves. Although not all varicoceles are associated with impaired spermatogenesis, their presence has been consistently linked to decreased sperm quality, altered testicular function, and oxidative stress [3,4].

Surgical repair is recommended in infertile men with clinical varicocele, abnormal semen parameters, and otherwise unexplained infertility, and in adolescents with a persistent small testis and a size difference greater than 2 ml or 20%, documented on two consecutive evaluations six months apart [5]. Common techniques include open (Palomo, Ivanissevich), microsurgical subinguinal, and laparoscopic varicocelectomy, each with distinct advantages and limitations. The microsurgical technique remains the gold standard due to its low complication and recurrence rates, though it is technically demanding and not universally available [6].

In recent years, the advent of three-dimensional (3D) laparoscopic imaging has introduced a new level of visual precision in minimally invasive surgery. Unlike traditional two-dimensional systems, 3D laparoscopy offers enhanced depth perception, spatial orientation, and more accurate tissue dissection, contributing to shorter learning curves and potentially better surgical outcomes [7]. While widely adopted in urology for procedures such as prostatectomy and nephrectomy, its role in varicocelectomy remains underexplored. Given its visual advantages, 3D laparoscopy may allow for microsurgery-like precision, particularly in identifying and preserving critical structures such as the testicular artery and lymphatics, without the need for adjuncts such as Doppler or optical magnification.

The aim of this study was to evaluate the safety and efficacy of 3D laparoscopic varicocelectomy in patients with ultrasound-confirmed varicocele and abnormal semen parameters, by analyzing operative and postoperative outcomes, patient satisfaction, and changes in semen parameters before and six months after surgery.

## Materials and methods

Study design 

This study is a retrospective analysis of prospectively collected data from a single center, including male patients diagnosed with varicocele who underwent 3D laparoscopic varicocelectomy between February 2021 and April 2025. The study was conducted at Lefkos Stavros - the Athens Clinic, in Athens, Greece. The study was conducted according to the guidelines of the Declaration of Helsinki. Written informed consent was obtained from all individual participants included in the study. According to the regulations of our institute, institutional review board (IRB) approval was not required for this retrospective study and was therefore waived.

Study population

Inclusion criteria were patients aged 18-45 years with ultrasound-confirmed (Doppler) varicocele, abnormal semen parameters and/or testicular discomfort, and a minimum follow-up of six months. Exclusion criteria included previous varicocele repair, azoospermia, testicular atrophy, or identifiable alternative causes of infertility (e.g., hypogonadism, genetic syndromes, obstruction). The sampling technique was consecutive sampling of all patients who met the predefined inclusion and exclusion criteria and underwent surgery in our institute during the study period.

Surgical technique

All procedures were performed by the same experienced surgeon using a standardized 3D transperitoneal laparoscopic approach. Under general anesthesia, three ports were inserted (one 10 mm umbilical trocar and two 5 mm lateral trocars). After mobilization of the sigmoid colon (left-sided cases), the internal spermatic vessels were isolated, and the veins were clipped and divided. The testicular artery was meticulously identified and preserved through visual dissection only, without the use of intraoperative Doppler or magnification systems. Lymphatics were spared where visible, but no methylene blue dye was applied. No drains were placed. All patients were discharged on the same or the following day. Pain was assessed using a 10-point Visual Analogue Scale (VAS) at 24 and 48 hours postoperatively. Follow-up visits were conducted at six weeks and six months and included clinical examination for recurrence or hydrocele, scrotal Doppler ultrasonography, and semen analysis according to the WHO 2021 criteria [8].

Outcome measures

Primary outcomes included operative time, complication rates (intraoperative and postoperative), and recurrence rate. Secondary outcomes included postoperative pain, patient-reported satisfaction, and changes in semen parameters (concentration, motility). Continuous variables are presented as mean ± standard deviation (SD), and categorical variables are presented as counts and percentages.

## Results

A total of 34 patients underwent 3D laparoscopic varicocelectomy during the study period. The mean patient age was 31.2 ± 6.1 years. The majority of cases were left-sided varicoceles (n = 24, 70.6%), with bilateral repair performed in 10 patients (29.4%). All varicoceles were diagnosed via scrotal Doppler ultrasound. The mean maximum vein diameter on scrotal ultrasound was 3.17 ± 0.52 mm.

Operative and postoperative outcomes

The mean operative time was 22.3 ± 2.4 minutes for unilateral cases and 28.6 ± 3.1 minutes for bilateral cases. No intraoperative complications occurred, and all cases were completed without the need for conversion to open surgery. Postoperative hydrocele formation and testicular atrophy were not observed. During the six-month follow-up, there was one case of recurrence (1.85%).

Postoperative pain and patient satisfaction

The mean VAS score at 24 hours was 1.2 ± 0.9, decreasing to 0.6 ± 0.7 at 48 hours. No patient required opioids after the first 24 hours. At the six-week follow-up, patient-reported satisfaction was assessed, with 70.6% (n=24) of patients rating their experience as “Excellent”, 23.5% (n=8) as “Very good”, and 5.9% (n=2) as “Moderate”.

Semen analysis outcomes

Among the 34 patients with available pre- and six-month postoperative semen analyses, the mean sperm concentration increased from 15.1 ×10⁶/mL to 34.3 ×10⁶/mL (p < 0.01), progressive motility improved from 28.4% to 44.9% (p < 0.01), and total motile sperm count (TMSC) improved from 5.1 ×10⁶ to 17.4 ×10⁶ (p < 0.01). The surgical and functional outcomes of 3D laparoscopic varicocelectomy are presented in Table 1.

**Table 1:** Surgical and functional outcomes of 3D laparoscopic varicocelectomy (N=34) Continuous variables are presented as mean ± standard deviation (SD), and categorical variables are presented as counts and percentages. VAS: Visual Analogue Scale

Parameters	Values
Age (years), mean ± SD	31.2 ± 6.1
Left-sided varicocele, n (%)	24 (70.6%)
Bilateral repair, n (%)	10 (29.4%)
Maximum diameter of veins (mm), mean ± SD	3.17 ± 0.52
Operative time - unilateral (minutes), mean ± SD	22.3 ± 2.4
Operative time - bilateral (minutes), mean ± SD	28.6 ± 3.1
Intraoperative complications	0
Postoperative hydrocele	0
Recurrence at 6 months, n (%)	1 (1.85%)
Testicular atrophy	0
VAS score at 24 hours, mean ± SD	1.2 ± 0.9
VAS score at 48 hours, mean ± SD	0.6 ± 0.7
Satisfaction: Excellent, n (%)	24 (70.6%)
Satisfaction: Very good, n (%)	8 (23.5%)
Satisfaction: Moderate, n (%)	2 (5.9%)

## Discussion

This study demonstrates that 3D laparoscopic varicocelectomy can be performed with safety, short operative time, and significant improvement in semen parameters, suggesting its potential as a modern alternative to microsurgical repair. The introduction of three-dimensional imaging systems in laparoscopic surgery has significantly improved visual clarity, spatial orientation, and tissue discrimination. Compared to standard 2D laparoscopy, 3D vision offers magnified depth perception, allowing precise anatomical dissection [7]. In our series, these advantages enabled clear identification of the testicular artery and complete skeletonization of the gonadal vein, a critical step for minimizing recurrence [9]. This skeletonization technique, which involves the careful separation of the gonadal vein from adjacent structures under direct vision, helps ensure the ligation of all venous branches, including lateral collaterals and small tributaries, without the need for Doppler or vital dyes. This is especially important given that incomplete vein control is a well-known risk factor for varicocele recurrence, particularly in laparoscopic approaches [1,9].

Our findings compare favourably with published series. A meta-analysis by Cayan et al. reported a recurrence rate of 4.3% and a hydrocele rate of 2.84% in laparoscopic varicocelectomy, while microsurgical techniques had recurrence and hydrocele rates of 1.05% and 0.44%, respectively [2]. Additionally, sperm concentration and progressive motility improved significantly after 3D laparoscopic varicocelectomy, consistent with prior reports supporting the role of varicocele repair in improving fertility parameters [10]. The mean operative time in our study (22.3 minutes) was substantially shorter than the reported averages for both laparoscopic (34 ± 10 min) and microsurgical (62 ± 17 min) varicocelectomy, demonstrating the potential for procedural efficiency without compromising quality [11]. Postoperative pain was minimal, and patient satisfaction was high, outcomes that support the effectiveness of the procedure from the patients’ perspective. The comparison of varicocelectomy techniques based on the available literature is presented in Table 2.

**Table 2:** Comparison of varicocelectomy techniques based on literature VAS: Visual Analogue Scale

Parameter	Microsurgery[2,11,12]	Laparoscopy[2,11,12]	3D Laparoscopy (Current Study)
Recurrence rate	1.05%	4.3%	1.85%
Hydrocele rate	0.44%	2.84%	0%
Operative time, mean ± SD	62 ± 17 minutes	34 ± 10 minutes	22.3 ± 2.4 minutes
Need for Doppler/dye	Yes	Occasional	No
Artery identification	High	Variable	High
Postoperative pain (VAS 24 hours), mean ± SD	5.2 ± 1.14	0.7 ± 0.82	1.2 ± 0.9
Patient satisfaction	High	Moderate–High	High

Limitations and strengths of the study

However, this study is not without limitations. Potential confounding factors include its retrospective design, patient selection, unmeasured lifestyle or fertility variables, and the absence of a control group, all of which can independently influence the study's outcomes. The absence of a comparative arm limits the study’s ability to demonstrate the superiority or equivalence of 3D laparoscopic varicocelectomy relative to other techniques, although an indirect comparison based on the available literature is provided. The small sample size may reduce the statistical power of the study for assessing certain outcomes, particularly relatively rare complications such as hydrocele and testicular atrophy. Furthermore, the follow-up duration of six months is likely too brief to fully evaluate recurrence and fertility outcomes. Certain limitations inherent to the transperitoneal laparoscopic approach should also be acknowledged. The need for intra-abdominal access and peritoneal closure carries potential risks, such as intestinal, vascular, and nerve damage, or peritonitis, and may be less desirable in select populations (e.g., pediatric patients or those with prior abdominal surgery). Complications related to pneumoperitoneum should also be mentioned, for example, the risk of pulmonary embolism, pneumo-scrotum, and postoperative pain in the right shoulder. Additionally, the division of the gonadal vein proximally at the level of the internal ring, although anatomically efficient, can miss low-lying external spermatic or cremasteric collaterals, a potential source of recurrence [9].

Nevertheless, this study’s strengths include mitigation of inter-surgeon variability by analyzing data from a single surgeon, the use of a standardized surgical approach for all patients, the exemption from mandatory use of adjunctive Doppler or vital dyes, and the assessment of the patient-reported surgical experience, supporting its merit in evaluating a novel method for varicocele repair. It is important to emphasize that no technique has consistently proven superior across all domains. While microsurgery remains the gold standard in many centers, 3D laparoscopy may offer a comparable alternative, particularly where microsurgical expertise or equipment is not available.

## Conclusions

Three-dimensional laparoscopic varicocelectomy, through enhanced anatomical visualization, fast dissection, and complete gonadal vein skeletonization, may be considered a robust alternative to microsurgical varicocelectomy, especially when performed by experienced laparoscopic surgeons. Future randomized studies comparing 3D laparoscopic varicocelectomy with microsurgical repair in terms of fertility outcomes and cost-effectiveness are warranted.
